# EVAE-Net: An Ensemble Variational Autoencoder Deep Learning Network for COVID-19 Classification Based on Chest X-ray Images

**DOI:** 10.3390/diagnostics12112569

**Published:** 2022-10-22

**Authors:** Daniel Addo, Shijie Zhou, Jehoiada Kofi Jackson, Grace Ugochi Nneji, Happy Nkanta Monday, Kwabena Sarpong, Rutherford Agbeshi Patamia, Favour Ekong, Christyn Akosua Owusu-Agyei

**Affiliations:** 1 School of Information and Software Engineering, University of Electronic Science and Technology of China, Chengdu 610056, China; 2 Department of Computing, Oxford Brookes College of Chengdu University of Technology, Chengdu 610059, China; 3 School of Computing and Artificial Intelligence, Southwest Jiaotong University, Chengdu 610032, China

**Keywords:** autoencoder, variational autoencoder, ensemble learning, deep learning, latent embedding

## Abstract

The COVID-19 pandemic has had a significant impact on many lives and the economies of many countries since late December 2019. Early detection with high accuracy is essential to help break the chain of transmission. Several radiological methodologies, such as CT scan and chest X-ray, have been employed in diagnosing and monitoring COVID-19 disease. Still, these methodologies are time-consuming and require trial and error. Machine learning techniques are currently being applied by several studies to deal with COVID-19. This study exploits the latent embeddings of variational autoencoders combined with ensemble techniques to propose three effective EVAE-Net models to detect COVID-19 disease. Two encoders are trained on chest X-ray images to generate two feature maps. The feature maps are concatenated and passed to either a combined or individual reparameterization phase to generate latent embeddings by sampling from a distribution. The latent embeddings are concatenated and passed to a classification head for classification. The COVID-19 Radiography Dataset from Kaggle is the source of chest X-ray images. The performances of the three models are evaluated. The proposed model shows satisfactory performance, with the best model achieving 99.19% and 98.66% accuracy on four classes and three classes, respectively.

## 1. Introduction

In late December 2019, the COVID-19 disease was discovered in Wuhan, a city in eastern China, and began spreading worldwide. By March 2020, the World Health Organization (WHO) classified it as a pandemic. COVID-19 is a pathogen strain caused by SARS-CoV2 that causes severe acute respiratory discomfort and exhibits symptoms of pneumonia in humans. The infection usually starts in the mucous membrane in the throat and rapidly spreads to the lungs. Once in the lungs, it impairs function and mutates rapidly before a patient can be diagnosed correctly. It is transmitted efficiently from human-to-human via aerosol and is highly infectious. This makes COVID-19 a public health emergency. Early detection and diagnosis are significant for breaking the transmission chain and controlling its spread.

To help control the spread of the disease, healthcare professionals and researchers have adopted several modalities to detect the virus. Some of the modalities include reverse transcriptase–polymerase chain reaction (RT-PCR) test [1], chest X-ray (CXR) image [2,3], and computed tomography (CT) scans [4]. RT-PCR has been the go-to diagnostic modality for detecting COVID-19 pathogens. It requires acquiring a respiratory specimen from the subject’s body. Though efficient, it has some disadvantages, such as a longer detection time and a lower detection rate.

Chest radiography images in the last decade have been used as a primary diagnostic tool in clinical practice to examine the cardiothoracic region for abnormalities. Pulmonary fibrosis, pneumonia, emphysema, chronic bronchitis, and lung cancer are a few of the pulmonary diseases that have been detected using chest radiography images [5,6]. In using CXR images as a diagnostic tool, lung abnormalities, such as bilateral or interstitial lung consolidation and/or ground grass opacities [7] can be identified, making CXR images a remarkable tool for detecting of COVID-19 disease at the early stage [8]. Given the similarity between COVID-19 and other respiratory disorders, such as pneumonia, the experience of the specialist is essential to identify and characterize COVID-19 biomarkers from other related clinical findings. Manually characterizing and identifying these biomarkers is tedious and does not permit the procedure to be repeated since it is time-consuming. Therefore, an automatic system is required to identify normal or COVID-19 cases with high accuracy by analyzing CXR images. Not only will it speed up the detection process, but it will also help reduce the workloads of health professionals.

Detecting COVID-19 is a typical classification problem in machine learning (ML) and deep learning (DL). ML and DL have demonstrated their effectiveness in solving problems in several domains. One advantage that has placed ML and DL as benchmark techniques in medical imaging is their computational capabilities and the availability of large labeled datasets [9]. They have been explored to detect pulmonary abnormalities in CXR imaging [10]. A deep convolutional neural network (CNN) for classifying lung images was proposed by Anthimopoulos et al. [11]. A CNN-based method to quantify the percentage of emphysema on simulated CXR images was presented by Campo et al. [12]. A deep neural network that incorporates both global and local features to identify pneumonia was proposed by Jaiswal et al. [13]. In the context of COVID-19, several works have been proposed to detect COVID-19 disease using CXR images [14,15,16,17].

DL models may suffer from overfitting, high variance, and/or generalization errors because of the limited size of the training data and the presence of noise in the training data. Ensemble learning is a technique used to combine multiple models and is a practical approach to handling these errors. Not only does it handle these errors, it also yields better results compared to single models [18].

Researchers have recently found that unsupervised pre-training helps train supervised deep neural networks [19]. One such fundamental unsupervised method employed to transform raw input data into a meaningful representation is autoencoders (AEs) [20]. Unlike conventional AEs, which try to learn a distance function, VAEs, proposed by Kingma and Welling [21], are powerful generative AEs that provide high-quality representations of the raw input data and generate desirable virtual instances in the controllable smooth latent space [22].

Compared with conventional AEs, VAEs are highly capable of a more general approximation of the intractable posterior density and can efficiently carry out the inference [21]. These advantages combined make VAEs extensively utilized and successful for a variety of machine learning tasks such as audio data recognition [23], text captioning [24], and natural image processing [25]. Furthermore, the latent representations generated by VAEs can be controlled more accurately owing to the variational lower bound in the optimization of VAEs. This accurate modeling could provide superior representations compared to other AE variants and enhance the results of several downstream tasks, such as image classification [26].

Additionally, by adjusting the distribution parameters, the generation of new instances can be easily controlled since VAEs learn the parameters of the specific probabilistic distributions. Again, due to their closed-form objective function, VAEs are more stable throughout training stages than other generative paradigms, such as generative adversarial networks (GANs) [27], and have the potential to generate shaper samples that are comparable to GANs [28]. Another major benefit of VAEs is their capability to handle larger datasets compared to variational inference. This is because VAEs can operate quickly using a single feedforward neural network to represent a stochastic function of the input variables. In contrast, variational inference typically becomes increasingly computationally complex as the number of samples increases.

Motivated by this, we propose EVAE-Net, an ensemble variational autoencoder deep learning network that combines the high-quality latent representations generated by VAE and ensemble learning for COVID-19 classification based on chest X-ray images. Three variants of EVAE-Net are proposed, and their performances are compared. The proposed model consists of two encoders, one or two reparameterization phases, a classification head, and one or two decoders. In the case of the single reparameterization phase, the feature maps from the encoders are merged before sampling the latent embeddings. The latent embeddings are then passed to the classification head and the decoder for reconstruction. In the case where each encoder has its reparameterization phase, the feature maps from each encoder are passed to their respective reparameterization phase to generate the latent embeddings. The latent embeddings are merged to form a single latent embedding and passed to the classification head. The individual latent embeddings are passed to their respective decoders for reconstruction. Our proposed methodology achieves promising classification performance, with the best model achieving 98.66% accuracy, 98.47% recall, 98.60% F1 score, and 98.75% precision for three classes, and 99.19% accuracy, 98.82% recall and precision, and 98.94% F1 score for four classes.

### Contributions

The main contributions of our work are summarized as follows:

- Propose a deep learning model based on VAE and ensemble learning for COVID-19 classification. Particularly, three models are proposed. Each model consists of an encoder for feature extraction, a reparameterization phase for sampling latent vectors from the extracted features, a decoder for reconstructing the input image from the latent vector, and a classification head for classifying COVID-19.
- Demonstrate the superiority of the proposed model by performing essential experiments. The experiment was conducted on both three classes and four classes using the standard COVID-19 radiography database. Several classification metrics were used, including accuracy, recall, precision, F1 score, and ROC-AUC. The experimental results demonstrate that the proposed EVAE-Net can automatically classify COVID-19 infections from CXR images and achieve better performance.
- Compare the performance of the proposed model to that of several state-of-the-art models, showing our proposed model outperforms these existing models.

The remaining work is structured as follows: In Section 2, we present related studies and works. Section 3 describes the research methodology. The datasets used in this work, evaluation metrics, and experimental setup are described in Section 4, followed by results and analysis in Section 5. Finally, we present discussion and conclusions of the study in Section 6 and Section 7, respectively.

## 2. Related Work

Since the outbreak of COVID-19, the scientific community has responded with high volumes of research dedicated to different levels. ML and DL models have been adopted in several areas to help deal with COVID-19 disease discovery and spread monitoring [29,30,31,32], prognosis [33], etc. Nevertheless, most of these ML and DL models focus on preprocessing X-ray or CT scan images to detect and classify COVID-19. Several tools, techniques, and datasets have been utilized to facilitate the detection and classification of COVID-19.

### 2.1. Deep Learning Models

Researchers have identified several approaches for identifying COVID-19 from chest X-ray images. One well-established method is the conventional method. However, this approach is not acceptable in the medical field due to its limitations, such as wasting time extracting features and inaccurate results leading to false positive results. In recent years, researchers have conducted several studies to detect COVID-19 infections. The researchers in [34] presented CovXNet, a deep learning model to classify COVID-19 and pneumonia infections by utilizing a public dataset containing 1493 non-COVID pneumonia and 305 COVID-19 pneumonia cases. Their model achieved an accuracy of 96.9%. Using four pretrained models, VGG16, ResNet50, DenseNet-121, and MobileNet, Umair et al. [35] proposed a technique for binary classification of COVID-19 and compared the performances of the four models. DenseNet-121 achieved the best performance with an accuracy of 96.49%.

The authors of Li et al. [36] proposed COVNET, a deep learning model for detecting COVID-19 infections. The proposed model successfully differentiated between COVID-19 pneumonia and community-acquired pneumonia (CAP) with sensitivity and specificity rates of 90% and 96%, respectively. A novel deep learning model, CovidDetNet, for detecting COVID-19 infections using chest radiograph images was proposed by Ullah et al. [37]. The proposed model comprises nine convolutional layers, one fully-connected layer, two activation functions (ReLU and Leaky ReLU), and two normalization operations (batch normalization and cross-channel normalization) and achieved an accuracy of 98.40%. A 17-layered deep learning model, DarkCovidNet, with different filter sizes for detecting COVID-19 infection, was presented by Ozturk et al. [38]. The authors used DarkNet [39] as the backbone for their model. The proposed model provided accurate diagnostics for binary classification (COVID vs. no findings) with 98.08% accuracy and multi-class classification (COVID vs. no findings vs. pneumonia) with 87.02% accuracy.

The authors of Khan et al. [40] proposed CoroNet, a CNN for the classification of COVID-19 by modifying an Xception pre-trained model. The model achieved an accuracy of 89.6%. In Agrawal and Choudhary [41], researchers presented FocusCovid, an automated deep learning model for detecting COVID-19 using chest radiograph images. The authors used FocusNet [42], a UNet-based encoder–decoder architecture proposed for medical image segmentation, as their backbone network. The model achieved 99.2% and 95.2% accuracy for binary classification and multi-classification, respectively. In [43], a convolution support estimation network (CSEN) that combines the advantages of a representation-based technique and deep learning was presented to detect COVID-19 infections from X-ray images. The proposed method uses training samples and a dictionary to map the sparse support coefficients to the query samples.

Other works have also exploited automated feature extraction and filtering techniques [44,45,46,47]. For example, the authors of [44] segregated COVID-19 positive cases and healthy cases by exploring several image filter techniques, such as a conservative smoothing filter and a Gaussian filter, and feature extraction techniques, such as linear discriminant analysis (LDA) and principal component analysis (PCA). The extracted features were then passed to various classification models, including CNN, SVM, and logistic regression (LR). The proposed model achieved impressive results by attaining an overall accuracy of 99.93%.

### 2.2. Transfer Learning Methods

In the COVID-19 detection and classification literature, most of the ML and DL models employed use pre-trained models as the baseline of their models. The pre-trained models were trained using the Imagenet dataset [48], and their weights are available for download. Although the baseline models used were the same, the architecture of the individual models is different [49]. VGGNet [50], ResNet [51], EfficientNet [52], DenseNet [53], and SqueezeNet [54] are few examples of state-of-the-art pre-trained models. In the literature, a large number of works have been proposed using transfer learning [49,55,56,57,58,59,60,61,62,63]. The authors of [62] implemented a deep learning model by fine-tuning three pre-trained models—VGG16, VGG19, and ResNet201—using chest X-ray images for binary classification of COVID-19. Using Bi-LSTM and CNN, Aslan et al. [60] designed a hybrid model to detect COVID-19 from CT scans. The authors used AlexNet as their pre-trained models. Similarly, Apostolopoulos and Mpesiana [49] proposed a CNN architecture based on transfer learning using ReLU [64] as the activation function and dropout to deal with overfitting. A binary and multi-class classification model using NASNet-large, DenseNet169, InceptionV3, ResNet18, and Inception ResNetV2 was proposed by Punn and Agarwal [59]. To overcome class imbalance, the authors used a weighted class-loss function (giving higher weight to COVID labels) and a random sampling technique (upsampled minority class). Using transfer learning and fine-tuning, El Asnaoui et al. [65] conducted a comparative study on several pre-trained models. They applied intensity normalization [66] during the preprocessing stage to enhance image quality, and they applied contrast-limited adaptive histogram equalization (CLAHE) [67]. Similarly, using five pre-trained models, the authors of [63] presented a deep learning model to detect COVID-19 from CT scan images. The authors assessed the performance of histogram equalization (HE) and CLAHE enhancement on CT scan images. The best pre-trained model attained an accuracy of 95.75%.

### 2.3. Autoencoders and Variational Autoencoders

In recent years, autoencoders (AEs) and variational autoencoders (VAEs) have been exploited for different medical tasks [68,69,70,71,72,73,74]. Given input data, AEs and VAEs transform the input from a high dimension to a low dimension known as a latent vector. Due to this capability, they have also been adopted for dimensionality reduction tasks [75,76,77]. VAEs are probabilistic variations of AEs. Unlike AEs, which attempt only to learn the latent representation of the input data, VAEs attempt to learn the latent distribution of the input data [78]. AE and VAE approaches have been adopted in several works. The authors of Rashid et al. [79] proposed a two-stage deep CNN scheme dubbed “AutoCovNet” to detect COVID-19. They used an encoder–decoder at the first stage and an encoder-merging network for the second stage. The weights learned from the first stage are used to initialize the encoder in the second stage. The outputs from the different layers in the encoder model are connected to the encoder-merging network, which finally performs feature extraction. The extracted features are then used to perform classification. An integrated model consisting of a pre-trained model, sparse autoencoder, and a feedforward neural network was proposed by J.L. et al. [80]. Similarly, Abdulkareem et al. [81] proposed a model that used stacked autoencoders instead of sparse autoencoders. Other works have also explored stacked autoencoders [82,83,84]. To deal with anomaly localization and lack of pixel annotation in CT images, Zhou et al. [78] proposed a “Weak Variational Autoencoder for Localisation and Enhancement (WAVLE)” framework with two parts: the localization part, which generates attention maps by combining a context-encoding variational autoencoder with a gradient-based technique; and an enhancement part that localizes the infected regions in the CT images, combining the attention maps generated in the first part. An unsupervised deep learning variational autoencoder model was proposed by Mansour et al. [85]. InceptionV4 with Adagrad was used as the feature extractor, and an unsupervised VAE performed classification. The quality of the image was enhanced using adaptive Wiener filtering during preprocessing. So far, this is the only work similar to our work. In our work, we explore the latent representations generated by VAE and leverage the capabilities of ensemble learning to design three ensembled variational autoencoder models to classify COVID-19. Several other methods and techniques to detect and classify COVID-19 have been proposed in the literature [15,17,86,87,88,89,90,91,92]. Table 1 summarizes the related works.

The abovementioned studies use convolutional operations to extract relevant features from the given input. The feature map is then passed to the classification head to detect or classify COVID-19. These feature maps may contain the relevant features extracted from the input image. However, a convolution neural network sees the input image as a cluster of pixels arranged in distinct patterns. It does not understand them as components present in the image nor the probability distribution of these components. With VAE, given a feature map, it describes the probability distribution of the feature map in a latent space. Thus, each attribute in the feature map is represented as a probability distribution for which we can randomly sample latent vectors for classification instead of using the entire feature map. This statistical distribution enforces a continuous, smooth latent space representation of the latent vector with its values near one another in a latent space, thereby acting as further filtering of the feature map to a reduced dimension but still holding relevant features with a probability distribution for classification.

## 3. Materials and Methods

This section discusses the theoretical aspect of VAEs and our proposed model. We describe the theory of variational autoencoders and the loss functions used in this work. We then describe the architectural design of the three proposed approaches.

### 3.1. Variational Autoencoders

**Figure 1:**
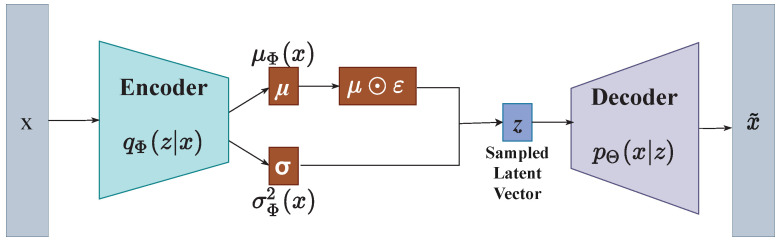
Basic architecture of a variational autoencoder.

AEs are neural network architectures with two main parts: an encoder $f_{e}$ and a decoder $f_{d}$. Given input datum $x_{i}$, the encoder transforms $x_{i}$ into a latent representation $z_{i}$, which is of lower dimension than $x_{i}$. The decoder takes $z_{i}$ as input and attempts to reconstruct $\tilde{x_{i}}$. Reconstruction loss, L, is computed to measure the model’s performance. L is computed by comparing the difference between $x_{i}$ and $\tilde{x_{i}}$. The weights of the model are updated through backpropagation. VAEs are probabilistic variations of AEs that combine Bayesian variational inference and deep learning. Similar to AEs, VAEs also have encoders and decoders and perform the same function. Figure 1 shows the basic architecture of VAEs. Instead of just learning the latent representation, VAEs learn the probability distribution of the training data through an amortized inference procedure that provides the means for computing the latent representation of the input data via the encoder. The encoder acts as the variational posterior $q_{\phi }\left(z|x\right)$, while the decoder acts as a generative model representing the likelihood $p_{\theta }\left(x|z\right)$. Given the posterior and the likelihood, we can define a joint inference distribution as

(1) $$q_{\phi }\left(z,x\right)\equiv p_{\theta }\left(x\right)q_{\phi }\left(z|x\right)$$

Any probability distribution function (PDF) can be used as the posterior distribution, but it is mainly assumed to be a multivariate Gaussian with diagonal covariance matrix $p_{z}\left(z\right)=N\left(z;0,I\right)$. The likelihood function $p_{\theta }\left(x|z\right)$ can be a Gaussian distribution or a Bernoulli distribution. In the encoder, the weights and biases are parameterized by the variational parameter ϕ, while in the decoder, the weights and biases are parameterized by the model parameter θ. Unlike in autoencoders, where the encoder outputs *z* (a probability distribution representing the latent embeddings), encoders in VAEs output μ and $\log\left(\sigma^{2}\right)$ and generate ε from N(0,I), out of which *z* is sampled using a reparameterization trick. The variable *z* is then fed as input to the decoder, which tries to reconstruct *x*; *z* is defined as:

(2) $$z=g\left(\varepsilon ,\phi ,x\right)=\mu +\sigma ⊙\varepsilon$$

where ⊙ is an elementwise multiplication operation, and *z* is a probability distribution representing the relevant features from the input *x*. Assuming $z∼N\left(\mu ,\sigma^{2}\right)$, then *z* can be reparameterized by

(3) $$z=\mu +\sigma \varepsilon ,\varepsilon ∼p\left(\varepsilon \right)=N\left(0,1\right)$$

Since $p_{\theta }\left(z\right)$ and $q_{\phi }\left(z|x\right)$ are both Gaussian distributions, the difference between the two distributions can be computed directly. Given a data point $x_{\left(i\right)}$, the resulting likelihood is calculated as:

(4) $$\begin{array}{c} L\left(\theta ,\phi ;x\right)=-E_{q_{\phi }\left(z|x_{i}\right)}\left[\log p_{\theta }\left(x|z\right)\right]+\\ \;\;\;\;\;\;\;\;D_{KL}\left(q_{\phi }\left(z|x_{i}\right)\|p_{\theta }\left(z\right)\right) \end{array}$$

Equation (4) is known as evidence lower bound (ELBO). The objective of the model is to maximize ELBO L(θ,ϕ,x) with respect to θ, the model parameters, and ϕ, the variational parameters. The first part of ELBO represents the reconstruction loss, which ensures the decoder is able to reconstruct $\tilde{x_{i}}$ from the latent representation *z*. The second part of ELBO acts as a regularizer called a divergence. It measures the divergence between $q_{\phi }\left(z|x\right)$ and $p_{\theta }\left(z\right)$ as well as penalizes the entanglement between the components in the latent space.

### 3.2. Kullback–Leiber (KL) Divergence

KL divergence measures how similar two probability distributions are. Given two probability distributions p(x) and q(x) defined over *x*, the KL divergence denoted by $D_{KL}\left(p\left(x\right)||q\left(x\right)\right)$ from q(x) to p(x) is defined as:

(5) $$D_{KL}\left(q\left(x\right)||p\left(x\right)\right)=\sum_{i}\left(p\left(x_{i}\right)\log\left(\frac{p\left(x_{i}\right)}{q\left(x_{i}\right)}\right)\right)$$

### 3.3. Maximum Mean Divergence (MMD)

Similar to KL divergence, MMD measures how close two distributions are to each other. Given two probability distributions, p(x) and q(x), MMD, denoted as

$MMD\left(p\left(x\right)\|q\left(x\right)\right)$, is defined as:

(6) $$\begin{array}{c} MMD\left(q\left(x\right)\|p\left(x\right)\right)=E_{q(x),q(x)}\left[k\left(x,x^{{}^{\prime }}\right)\right]+\\ E_{p(x),p(x)}\left[k\left(x,x^{{}^{\prime }}\right)\right]-\\ 2E_{q(x),p(x)}\left[k\left(x,x^{{}^{\prime }}\right)\right] \end{array}$$

where $k(x,x^{{}^{\prime }})$ is any universal kernel, for which we use the Gaussian kernel in this work.

### 3.4. Loss Function Establishment

In this study, we adopted two objective functions. The first objective function ensures the VAE reconstructs ${\tilde{x}}_{i}$ as closer to the actual input datum $x_{i}$ such that the latent representation *z* that is created is in some particular distribution. First, we compute the reconstruction loss by measuring the difference between ${\tilde{x}}_{i}$ and $x_{i}$. The reconstruction loss, denoted as L(θ,ϕ;x), is defined as:

(7) $$L\left(\theta ,\phi ;x\right)=E_{q_{\left(\phi \right)}\left(z|x\right)}\left[\log p_{\theta }\left(x|z\right)-\log q_{\theta }\left(z|x\right)\right]$$

where $q_{\left(\phi \right)}\left(z|x\right)$ represents the latent distribution created by the encoder from the input data, and $p_{\theta }\left(x|z\right)$ represents the reconstructed distribution from the latent representation by the decoder. Afterwards, the divergence among the latent representation distribution is measured. In this study, we adopt the MMD. The final VAE loss is defined as:

(8) $$L_{VAE}=L\left(\theta ,\phi ;x\right)+MMD(q_{\phi }\left(z|x\right)||p\left(z\right))$$

The cross-entropy (CE) loss was adopted as the second objective function to handle the classification task. The CE loss function is computed as:

(9) $$L_{cls}\left(O,y\right)=-\frac{1}{N}\sum_{{}_{i=1}}^{N}y_{i}\log\left(S\left(O\right)\right)$$

where *N* represents the number of classes, $O\in R^{N\times 1}$ represents the output from the fully connected layer, *y* and $y_{i}$ represent the true label in the batch and predicted label of image *i* in the batch, respectively, and S is the softmax applied to the output of O to normalize it.

### 3.5. Ensemble Variational Autoencoder

This section details the architecture of our proposed model. Using VAE and ensemble learning, we propose three different architectures for the classification of COVID-19. Each architecture consists of two encoders (ResNet50 and VGG16), reparameterization, a classification head, and either one or two decoders. The classification layers of ResNet50 and VGG16 were removed.

#### 3.5.1. ResNet50 Encoder

The ResNet50 encoder follows the ResNet50 architecture [51]. The first layer is a convolutional layer with a kernel size of 7×7 and 64 different kernels with a 2×2 stride. The layer is immediately followed by a 2×2 max-pooling layer. Four sequential blocks follow Layer 1. Each block performs a convolution operation with different kernel sizes. In Block 1, there is a 1×1, 64 kernel convolutional operation followed by a 3×3, 64 kernel convolutional and finally a 1×1, 256 kernel operation. Block 1 is repeated 3 times, giving a total of 9 layers. Similarly, in Blocks 2, 3, and 4, the kernel sizes used are (128, 128, 512), (256, 256, 1024), and (512, 512, 1024), respectively. Blocks 2, 3, and 4 are repeated 4, 6, and 3 times, respectively, giving a total of 50 layers. Finally, an adaptive average pooling and flattening layer is applied to the output of Block 4.

#### 3.5.2. VGG16 Encoder

We adopted the VGG16 [50] architecture for the VGG16 encoder. There are two 3×3 convolutional filter layers and a 2×2 max-pooling layer. Each of these two layers are repeated 3 times. Next, there are three 3×3 convolutional filter layers and a 2×2 max-pooling layer, each of which is repeated two times. Finally, adaptive average pooling and flattening is applied.

#### 3.5.3. Reparameterization

The outputs from the encoders are transformed using a linear layer to the dimension of the latent space, $R^{B\times 128}$, where *B* is the batch size. We then perform random sampling from a standard normal Gaussian distribution having a mean and variance. Two linear layers are used to implement the mean and variance, which has the same dimension as the latent space. Standard deviation is computed from the variance, and Equation (2) was applied to sample *z*.

#### 3.5.4. Classification Head

The classification head takes *z* as input and performs a linear transformation on it. The output dimension of the classification head is $R^{B\times C}$, where *B* is the batch size, and $C=\left\{3,4\right\}$ is the number of classes.

#### 3.5.5. Decoders

The decoder performs the reverse operation of the encoder. It takes *z* as input and performs a ConvTranspose2d on it to upsample it to produce an output $\tilde{X}∼X$.

#### 3.5.6. Model One

**Figure 2:**
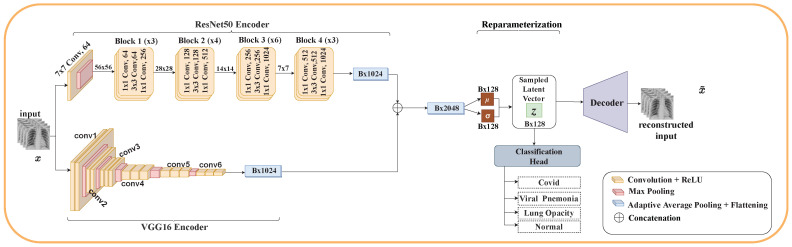
Model One: This architecture has two encoders and a single decoder. The feature maps of the last layers of both encoders are concatenated to give a richer feature map before reparameterization is done to sample the latent embedding *z*.

In this model, as depicted in Figure 2, the feature maps of the last layers in both encoders are concatenated before performing the reparameterization trick to sample the latent embedding *z*. This model allows us to merge at a high level the individual features from both encoders, giving us a richer feature map before sampling *z*. The sampled latent vector is then passed as input to the decoder to reconstruct the input and the classification head to classify the various types of pneumonia. In this model, only one decoder is used. There are two objective functions: $L_{VAE}$, which ensures the output of the decoder is closer to that of the input; and $L_{cls}\left(O,y\right)$, which provides the classification head the ability to accurately classify the various types of pneumonia. The advantage of this model is its simplicity. It takes advantage of concatenating the feature maps of each encoder before sampling the latent vector. Though this is the simplest of the three models, its performance depends on the sensitivity of how well each encoder can extract relevant features from the input since they share a single decoder and a single $L_{VAE}$ objective function.

#### 3.5.7. Model Two

**Figure 3:**
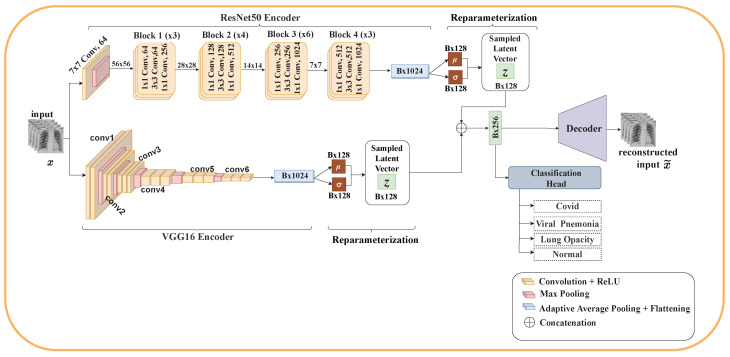
Model Two: Each encoder performs its own reparameterization to generate its own latent embedding *z*. The latent embeddings are then merged and passed as input to the decoder and classification head for reconstruction and classification of pneumonia, respectively.

This model combines separate low-level VAEs to form a high-level VAE. Each low-level VAE learns the representation of the input data to create its latent vector. These independent latent vectors are merged to create an integrated low-level representation. Like Model One, Model Two also employs a single decoder and two objective functions. The computational cost of this model is higher than that of Model One. There is a three-stage learning process involved: first, learning the low-level VAEs of individual encoders; second, reconstruction of the low-level representation to high-representation by the decoder; third, classification of the various types of pneumonia by the classification head. Despite its computational cost, it presents some advantages. The decoder and classification head input is composed of low-level representations from the individual encoders, which already have distribution regularization terms. Thus, the merged representations already consist of approximated multivariate standard normal distributions representing the input of each encoder. Similar to Model One, performance also depends on the sensitivity of how well each encoder can extract relevant features from the input, since they share a single decoder and a single $L_{VAE}$ objective function. Figure 3 depicts the architecture of Model Two.

#### 3.5.8. Model Three

**Figure 4:**
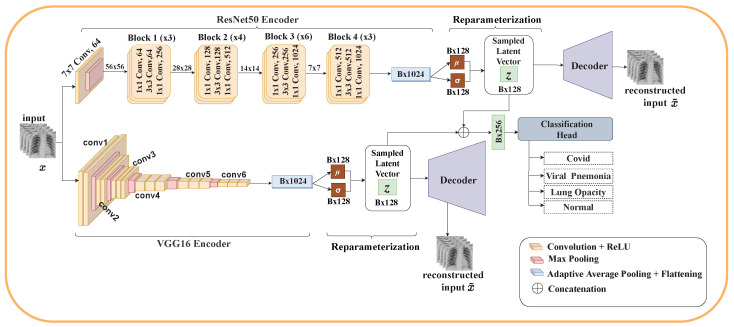
Model Three: Each encoder performs its own reparameterization to generate its own latent embedding *z*. The latent embeddings are passed to their respective decoders for reconstruction of the input image and then merged and passed as input to the classification head for classification of pneumonia.

This model is similar to Model Two. The difference is that for Model Three, each encoder implements its decoders. After reparameterization, the latent vectors of the individual encoder are merged to form one latent vector and then passed to the classification head. The individual latent vectors are then passed to their respective decoders to reconstruct the input. Three objective functions are used: two $L_{VAE}$ function—one for each encoder–decoder—and $L_{cls}\left(O,y\right)$ for the classification task. This model inherits all the advantages presented by architecture two. In addition, it solves the performance problem of Models One and Two since each encoder now implements its own decoder and $L_{VAE}$. Figure 4 depicts the architecture of Model Three.

## 4. Experiments

### 4.1. Dataset

**Figure 5:**
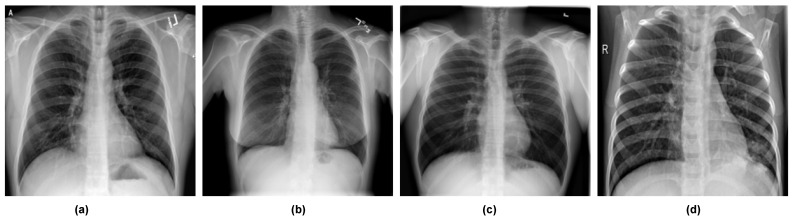
Sample datasets used in this work: (**a**) sample with COVID-19, (**b**) sample with lung opacity, (**c**) sample with normal, and (**d**) sample viral pneumonia.

In this study, we used the COVID-19 Radiography Database curated by [93,94], which is publicly available in the Kaggle repository. The dataset comprises 21,165 X-ray images: 3616 COVID-19-positive, 10,192 normal (non-COVID), 6012 lung opacity (non-COVID lung infection), and 1345 viral pneumonia. We experimented with both three and four classes. For four classes, we split the dataset into training (12,696 samples, representing 60% of the entire dataset), validation (6351 samples, representing 30% of the entire dataset), and testing (2118 samples, representing 10% of the entire dataset) sets. For the three classes, we combined lung opacity and viral pneumonia to form one set called viral pneumonia. To increase the number of COVID samples, we performed data augmentation using Augmentor to generate 6000 samples. We then sampled 5000 COVID images, 5000 normal images, and 5000 pneumonia images. We then split the dataset into training (10,499 samples, representing 70% of the dataset), validation (3750 samples, representing 25% of the dataset), and testing (750 samples, representing 5% of the dataset). Table 2 shows the composition of the dataset for four classes and three classes. Figure 5 shows samples of images from the dataset.

### 4.2. Selection of Backbone Networks

To select the backbone networks for our work, we experimented with four pre-trained models: ResNet50, DenseNet201, VGG16, and Xception, out of which we chose the top two models with the highest performance. We ran each model on the four-class dataset for 20 epochs using a learning rate of 0.00003 and the Adam optimizer. The ResNet50 pre-trained model obtained the best results, followed by VGG16. DenseNet201 and Xception showed similar results. Hence, we selected ResNet50 and VGG16 as the backbone networks for our model. Table 3 shows the results of the backbone network selection experiment.

### 4.3. Evaluation Metrics

In this study, five quantitative measures were adopted to measure the performance and effectiveness of our proposed model: accuracy, precision, recall, area under the curve (AUC), and F1 score.

TP (*True Positive*); TN (*True Negative*); FP (*False Positive*); FN (*False Negative*).

***Accuracy:*** Measures how close a predicted label is to the true label.
  (10) $$Accuracy=\frac{TP+TN}{TP+TN+FP+FN}$$
***Precision:*** Measures actual positives from all predicted positive labels:
  (11) $$Precision=\frac{TP}{TP+FP}$$
***Recall:*** Known also as sensitivity, measures the ratio between all positive samples correctly classified to all positive samples (both correct and incorrect). Recall shows a model’s ability to actually classify positive samples as positive.
  (12) $$Recall=\frac{TP}{TP+FN}$$
***ROC AUC Score:*** Shows the relationship that exists between the true positive rate (recall) and the false positive rate. It also shows how good a model is at differentiating positive and negative target classes. ROC AUC is calculated using area under the curve (AUC).
***F1 Score:*** Measures the harmonic mean of recall and precision.
  (13) $$F1Score=2\times \frac{Precision\times Recall}{Precision+Recall}$$
***Confusion Metric:*** Provides an intuitive and descriptive way to summarize the performance and correctness of a model.

### 4.4. Experimental Setup

In all the experiments, the input image was resized to a fixed size of 224×224 before feeding it to the network, there was a mini-batch size of 4 for both training and validation sets. The dimensions of the input image were 4×3×224×224. All models were trained for 20 epochs. For the validation set, the weight with the best accuracy was saved. We adopted two loss functions: maximum mean divergence (MMD) for variational autoencoder (refer to Equation (8)) and cross-entropy function for the classification task. The Adam optimizer was chosen as the primary optimizer and had a learning rate of 0.00003. We also experimented with three additional learning rates: 0.00001, 0.00002, and 0.00004. Table 4 shows the hyperparameters used in this study.

Choosing an optimal objective function for the experiment was also considered. Since the architectures involve multiple tasks—reconstruction and classification—the chosen objective functions should improve the disentanglement between the latent embeddings and the classification of the different types of pneumonia. For the encoder–decoder, the objective function should consider both the reconstruction loss and a regularization term. Mean square error (MSE) loss was chosen for the reconstruction task, with MMD as the regularization term and cross-entropy as the loss for classification.

The network weights were initialized randomly since we did not use pre-trained models as the backbone for our network. All experiments were conducted on a single GPU (GEFORCE RTX 3060) with an 8-core AMD Ryzen 7 3700X processor.

## 5. Results and Analysis

This section reports the various experimental results of the proposed models using the COVID-19 Radiography dataset. For each of the models, we conducted several experiments by changing some of the hyperparameters. For the initial experiment, the Adam optimizer with a learning rate of 0.00003 was used and was trained for 20 epochs with a batch size of 4. This was done for both three and four classes. In the subsequent experiments, the optimizer was kept constant while we varied the learning rate.

### 5.1. Model Complexity

Table 5 shows the computational complexity of our proposed model in terms of the number of parameters and multiply-and-accumulate operations (MACs). From the table, Models One and Two have almost the same complexity since both models share a single decoder unit. The slight difference between their complexities is a result of Model Two performing a reparameterization operation for both encoders compared to a single reparameterization operation in Model One. For Model Three, each encoder has its own decoder, resulting in a higher number of parameters. Additionally, there are two reconstruction loss functions for the reconstruction of the input image, and one cross-entropy loss for the classification task, resulting in higher MACs.

### 5.2. Validation and Testing Performance Evaluation

Table 6 shows the best performance metrics for each model on the validation set. From the table, it can be observed that a learning rate of 0.00003 produced better results for the four classes, while for the three classes, different learning rates produced different results for every model.

For three classes, Model One achieves 98.14% accuracy, 98% recall, 98.2% precision, and 98.15% F1 score; Model Two achieves 98% accuracy and 97.99% recall, precision, and F1 score; Model Three achieves 98.24% accuracy and recall, 98.32% precision, and 98.27% F1 score. For four classes, Model One achieves 96.44% accuracy, 95.18% recall, 96.48% precision, and 95.49% F1 score; Model Two achieves 97.12% accuracy, 96.18% recall, 96.23% precision, and 97.14% F1 score; Model Three achieves 98.72% accuracy, 98.52% recall, 98.55% precision, and 98.77% F1 score.

**Figure 6:**
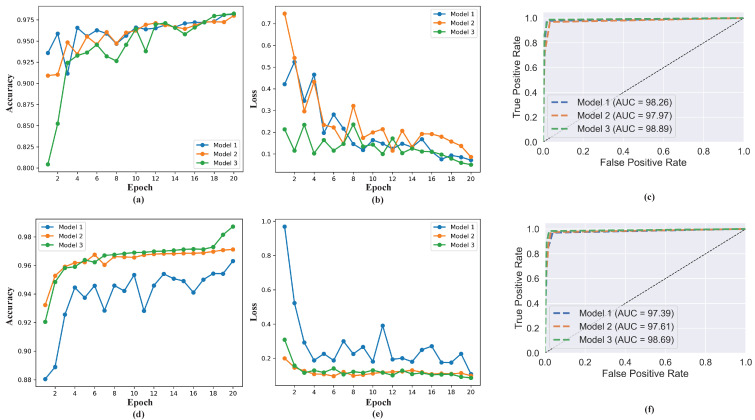
Performance comparison of the three models in terms of validation accuracy and loss. The (**a**–**c**) presents the performance of the three models on three classes, while the (**d**–**f**) presents the performance on four classes.

Table 7 shows the performance metrics of the best model (Model Three). For the three classes, it achieved 98.24% accuracy, recall, and F1 score, and 98.25% precision. For the four classes, an accuracy of 98.72%, recall of 98.52%, precision of 98.55%, and F1 score of 98.77% was achieved. Figure 6 shows the accuracy, loss, and ROC AUC graph comparing the performance of the three models for both three classes and four classes. These results indicates the proposed model’s capability in classifying COVID-19. For all the performances measured, the best results are obtained with 20 training epochs, which is an indication that the performance of the model increases gradually as the number of epochs increases.

We performed cross-validation using the testing set, as depicted in Table 8, which the model was not exposed to during training and validation. Cross-validations was performed to verify that our model does not suffer from overfitting and to test the model’s robustness. For three classes, Model One achieves 98.57% accuracy, 98.42% recall, and 98.47% precision and F1 score; Model Two achieves 98.43% accuracy, 98.37% recall, and 98.35% precision and F1 score; Model Three achieves 98.66% accuracy, 98.47% recall, 98.69% precision, and 98.60% F1 score. For four classes, Model One achieves 97.04% accuracy, 96.55% recall and F1 score, and 96.89% precision; Model Two achieves 97.99% accuracy, 97.90% recall, 97.70% precision, and 97.91% F1 score; Model Three achieves 99.19% accuracy, 98.82% recall and precision, and 98.94% F1 score. Table 9 and Table 10 show the confusion matrix for the cross-validation on the testing dataset. For three classes, out of a total of 487 radiographs, the model misclassified 130 radiographs; of those, 47 were COVID-19 images, 43 were normal images, and 40 were viral pneumonia images. For four classes, out of 2118 radiographs, the proposed model misclassified 17; of those, 4 were COVID-19 images, 3 were normal images, 6 were lung opacity images, and 4 were viral pneumonia images. This indicates the proposed model has higher true negative and true positive values and lower false negative and false positive values, suggesting the proposed model can accurately classify COVID-19 infections.

### 5.3. Ablation Studies

An ablation study was conducted to observe the contribution of the latent vector’s dimension to the model’s performance. The default dimension of the latent vector throughout the experiment was 128. Using a learning rate of 0.00003 and the Adam optimizer, we experimented with two lower dimensions for the ablation study: 32 and 64. The ablation study was conducted for both three classes and four classes on the validation dataset. Table 11 shows the ablation study for various latent sizes. The table shows that the model’s performance increases with an increase in the latent vector’s dimension. A latent dimension of 128 achieves higher performance in all the models.

### 5.4. Comparison with State-of-the-Art Methods

We compared our proposed model to various methods, as shown in Table 12. The comparison shows the proposed model, EVAE-Net, outperformed the methods in [37,40,41,95,96] using the same dataset (COVID-19 Radiography Database) for COVID-19 classification and methods that used other modalities [17,38,49,97]. It was worth noting that most of these methods only focused on either three classes or four classes. In our proposed model, we tested both three classes and four classes, making our model generalize better for COVID-19 classification.

## 6. Discussion and Future Work

In this study, we adopted VAE combined with an ensemble technique for the classification of COVID-19 with high accuracy. Table 4 and Table 7 show the best model’s validation and testing performance metrics. Of the three models, Model Three achieved the best performance. This reveals that the best result is obtained when each encoder implements its own decoder. With a single decoder, as with Model One and Model Two, the model’s performance highly depends on how each encoder can extract relevant features from the input, since they share a single $L_{VAE}$. With a decoder for each encoder, each encoder implements its $L_{VAE}$ with the advantage of learning to improve its performance from the loss by extracting more relevant features from the input. This further improves the latent embeddings sampled during the reparameterization stage since they are sampled from feature maps with more relevant features. A merger of the two latent vectors produces a richer latent vector, producing a higher classification result. The proposed EVAE-Net is more accurate than PCR since PCR results rely on the sample collection time and how they are stored and processed. Again, PCR results have high false negative rates when the patient is tested too early or too late after exposure to the virus. Further, the proposed model has higher true negative and true positive values and lower false negative and false positive values, suggesting the proposed model can accurately classify COVID-19 infections.

Although the proposed EVAE-Net produced interesting results, it still has some limitations, which we will address in future work. The proposed model cannot indicate the exact region of pneumonia in the chest X-ray image, which is vital for a radiologist. Future work will consider an attention mechanism focusing on the precise pneumonia region in the chest X-ray image. Since we only concentrated on using chest X-ray images for this study, the proposed model is biased toward chest X-ray images. This study did not consider other data modalities, such as CT scans. In future research, we will extensively examine other imaging modalities, which will help the model to generalize better.

Furthermore, we will combine several imaging modalities into a single dataset to investigate the robustness of the proposed model in future studies. We will also examine how image enhancement techniques such as discrete wavelet transform (DWT), CLAHE, etc., will enhance the feature maps from the encoders to improve the model’s performance. We believe images that the model incorrectly classified can be improved when enhanced.

Other limitations are associated with the nature of VAE architecture. VAE architecture requires rich domain-specific knowledge, and this barrier prevents end users without design expertise from utilizing VAEs. Even researchers with expert knowledge must go through an arduous trial-and-error process to tune the architecture manually. For example, finding the optimal depth of the VAE is unknown from the beginning. Thus, it is unknown how to choose the appropriate number of convolutional, pooling, and dense layers for the VAE architecture and what the optimal hyperparameters for each convolutional and deconvolutional layer are. In future studies, we will explore neural architecture search (NAS) to find solutions to this limitation.

In recent years, many Internet of Things (IoT) devices, particularly Medical Internet of Things (MIoT), have been adopted in the health sector to combat various medical issues. Several applications have been deployed in these smart devices for remote patient monitoring (RPM) and other related medical tasks. In fighting against COVID-19, one important aspect of containing the virus is an effective and fast diagnosis method. With the rate at which the virus spreads, diagnosing and screening it quickly is key to containing it. Therefore, there is a need to develop effective and efficient deep learning models leveraging the advantages of IoT and MIoT to diagnose and screen COVID-19 with speed.

## 7. Conclusions

This study proposed an ensemble variational autoencoder network for the classification of COVID-19 with high accuracy. We exploited variational autoencoders to produce feature-rich latent vectors for classification. Three different ensemble variational autoencoders were designed. Models One and Two have two encoders and a single decoder; Model Three has two decoders. In the case of Model One, the feature maps from the two encoders are concatenated before reparameterization to sample the latent vector. The latent vector is then passed to the classification head for classification and to the decoder for reconstruction of the input image. Models Two and Three each implement reparameterization. The latent vectors from reparameterization are merged and passed to the classification head and the decoder. Our model was trained on the COVID-19 Radiography Dataset. In the case of three classes, the best model achieved 98.66% accuracy, 98.47% recall, 98.60% F1 score, and 98.75% precision. For four-class classification, 99.19% accuracy, 98.82% recall and precision, and 98.94% F1 score were accomplished. We demonstrated that our model can automatically predict and classify COVID-19 by extracting relevant features from chest radiograph images. This will go a long way to help reduce radiologists’ workload and to avoid misdiagnosing COVID-19 patients. In future studies, we will adopt different DL strategies, such as attention mechanisms and NAS, to improve the computational complexity and performance of EVAE-Net.

## Figures and Tables

**Table 1:** Summary of related works.

Ref./Year	Technique	Advantages	Limitations
[34]/2020	Depthwise convolution with varying dilation rates	Efficiently extracts chest X-ray features by varying depthwise convolution and changing the dilation rate	No sensitivity analysis was performed
[35]/2021	Transfer learning	Classification task is made easier using pre-trained networks. Highlights the features extracted in the X-ray images using Grad-CAM. Dropout is used to avoid overfitting	Did not test the robustness of the model on multi-class classification
[36]/2020	CNN	Extracts both 2D local and 3D global representative features Vanishing gradient problem is solved by use of residual network	Model was trained on a smaller dataset
[37]/2022	CovidDetNet classifier	Computationally efficient due to fewer layers Successfully extracts more distinguishing features Normalization technique is used, which improves the convergence during training from the chest radiograph images	Did not test the robustness of the model on other classes, such as four-classes
[38]/2020	DarkCovidNet classifier	Classification performance is improved using 5-fold cross-validation	Could use dropout to avoid overfitting
[40]/2020	CoroNet classifier	Transfer learning is used to improve the quality of the deep features	Dropout can be used to avoid overfitting Model was trained on a small dataset
[41]/2021	FocusCovid classifier	Data augmentation and normalization are used to avoid overfitting Classification performance is improved using attention mechanisms Classification task is made easier using pre-trained networks. Cross-validation is used to estimate the general effectiveness of the models	Model was trained on a smaller dataset
[43]/2021	Convolution support estimation network (CSEN)	Efficient in terms of speed and memory usage	Performance degrades rapidly due to the scarcity of data
[59]/2021	Transfer learning	Class imbalance problem is dealt with using the weighted loss function Visualizes the prediction of the model using activation maps and LIME technique Cross-validation method is used to estimate the general effectiveness of the models	Focused only on posterior–anterior (PA) view of the X-rays, so unable to differentiate other views of X-ray images
[60]/2021	CNN-based transfer learning–BiLSTM	CNN combined with LSTM provides better classification accuracy Data augmentation used to avoid overfitting	Dropout can be used to avoid overfitting
[62]/2021	Transfer learning	A pre-trained network trained on a larger dataset is used to work efficiently on small datasets.	Model was trained on a small dataset. Only binary classification was performed.
[63]/2021	Transfer learning	HE and CLAHE were used to eliminate noise and improve the quality of the CT scan images. Data augmentation was applied to avoid overfitting. Pre-trained models are used to stabilize training	Model was trained on a small dataset. Only binary classification was performed.
[65]/2021	Transfer learning	Handles chest CT images and chest X-ray images. Intensity normalization CLAHE were used to eliminate noise and improve the quality of the X-ray images. Data augmentation, weight-decay, and L2 regularizer are applied over-fitting.	Only binary classification was done

**Table 2:** Dataset composition for both three and four classes.

	Set	COVID	Normal	Lung Opacity	Viral Pneumonia
Four classes	Training	2167	6115	3607	807
	Validation	1085	3058	1804	404
	Testing	364	1019	601	134
	**Total**	**3616**	**10,192**	**6012**	**1345**
Three classes	Training	3500	3500	-	3500
	Validation	1250	1250	-	1250
	Testing	250	250	-	250
	**Total**	**5000**	**5000**	**-**	**5000**

**Table 3:** Experimental results for the selection of the backbone network.

Pre-Trained Model	Accuracy (%)	Recall (%)	Precision (%)	F1 Score (%)
ResNet50	96.43	96.45	96.45	96.42
VGG16	96.21	96.19	96.21	96.24
DenseNet121	95.73	95.73	95.77	95.76
Xception	95.71	95.7	95.74	95.74

**Table 4:** Selected hyperparameters for EVAE-Net.

Hyperparameter	Value
Learning rate	0.00003
Optimizer algorithm	Adam
Number of epochs	20
Batch size	4
Latent dimension	128
Input image dimensions	3×224×224

**Table 5:** Computational complexity of the three models in terms of parameters (in millions) and multiply-and-accumulate operations (MACs).

Model	Parameters (M)	MACS (G)
Model One	56.176	92.291
Model Two	56.963	92.294
Model Three	87.745	123.120

**Table 6:** Best performance for the three models on the validation dataset. For each model, the performance metrics for both three classes and four classes are shown, along with the best learning rate.

Model	Predicted Classes	Learning Rate	Accuracy (%)	Recall (%)	Precision (%)	F1 Score (%)
Model One	three classes	0.00003	98.14	98.00	98.20	98.15
	four classes	0.00003	96.44	95.18	96.48	95.49
Model Two	three classes	0.00001	98.00	97.99	97.99	97.99
	four classes	0.00003	97.12	96.18	96.23	97.14
Model Three	three classes	0.00004	98.24	98.24	98.32	98.27
	four classes	0.00003	98.72	98.52	98.55	98.77

**Table 7:** Performance metrics of the best model among the three models for both three and four classes on the validation dataset.

Predicted Classes	Model	Accuracy (%)	Recall (%)	Precision (%)	F1 Score (%)
Three classes	Model Three	98.24	98.24	98.25	98.24
Four classes	Model Three	98.72	98.52	98.55	98.77

**Table 8:** Cross-validation results on the testing dataset for both three classes and four classes.

Model	Predicted classes	Accuracy (%)	Recall (%)	Precision (%)	F1 Score (%)
Model One	Three classes	98.57	98.42	98.47	98.47
	Four classes	97.04	96.55	96.89	96.55
Model Two	Three classes	98.43	98.37	98.35	98.35
	Four classes	97.99	97.90	97.70	97.91
Model Three	Three classes	98.66	98.47	98.75	98.60
	Four classes	99.19	98.82	98.82	98.94

**Table 9:** Confusion matrix for three classes on the testing dataset for the best model.

Predicted Class				
		COVID-19	Normal	Viral Pneumonia
**Actual Class**	COVID-19	203	7	40
	Normal	10	207	33
	Viral Pneumonia	25	15	210

**Table 10:** Confusion matrix for four classes on the test dataset for the best model.

		Predicted Classes			
		COVID-19	Normal	Lung Opacity	Viral Pneumonia
**Actual Class**	COVID-19	361	1	0	3
	Normal	1	1016	0	2
	Lung Opacity	2	1	595	3
	Viral Pneumonia	3	0	1	130

**Table 11:** Ablation study of different latent vector sizes on the performance of the model. Latent size of 128 achieved the best performance among all the models. (Optimizer = Adam; Learning rate = 0.00003; Epochs = 20; set = validation dataset).

Model	Latent Size	Accuracy (%)	Loss	Recall (%)	Precision (%)	F1 Score (%)
4 Classes						
**Model One**	32	93.12	0.2113	93.00	92.76	93.05
	64	94.52	0.1736	94.27	94.20	94.23
	**128**	**96.44**	**0.1105**	**95.18**	**96.48**	**95.49**
**Model Two**	32	94.79	0.1765	94.72	94.75	94.74
	64	95.27	0.1365	95.30	95.29	95.35
	**128**	**97.12**	**0.0998**	**96.18**	**96.23**	**97.14**
**Model Three**	32	95.88	0.1532	95.82	95.85	95.82
	64	96.27	0.1112	96.25	96.29	96.29
	**128**	**98.72**	**0.0875**	**98.52**	**98.55**	**98.77**
3 Classes						
**Model One**	32	95.46	0.1632	95.40	95.48	95.50
	64	96.32	0.1245	96.30	96.29	96.30
	**128**	**98.14**	**0.0966**	**98.00**	**98.20**	**98.15**
**Model Two**	32	95.19	0.1542	95.10	95.11	95.13
	64	96.35	0.1254	96.33	96.38	96.35
	**128**	**98.00**	**0.08061**	**97.99**	**97.99**	**97.99**
**Model Three**	32	95.46	0.1463	95.44	95.42	95.45
	64	96.65	0.1052	96.70	96.69	96.70
	**128**	**98.24**	**0.0506**	**98.24**	**98.32**	**98.27**

**Table 12:** Performance comparison of proposed model with existing studies. Methods with * used the COVID-19 Radiography Dataset.

Class	Method	Accuracy (%)	Recall (%)	Precision (%)	F1 Score (%)
**4**	Gopatoti and Vijayalakshmi [97]	94.00	95.31	95.31	93.74
	Ozturk et al. [38]	90.13	90.39	88.38	88.28
	* Khan et al. [40]	92.93	92.58	90.29	92.26
	Apostolopoulos and Mpesiana [49]	91.92	91.49	89.18	90.40
	Mostafiz et al. [17]	98.48	97.89	98.72	98.29
	**EVAE-Net**	**99.19**	**98.82**	**98.82**	**98.94**
**3**	Gopatoti and Vijayalakshmi [97]	97.05	96.96	94.44	95.38
	* Agrawal and Choudhary [41]	95.20	95.20	95.60	95.20
	* Wu et al. [95]	97.67	96.54	96.65	96.59
	* Aslan et al. [96]	96.29	96.42	96.42	94.53
	* Ullah et al. [37]	98.40	96.66	97.00	96.82
	**EVAE-Net**	**98.66**	**98.47**	**98.75**	**98.60**

## Data Availability

The dataset that supports this study was obtained from the Kaggle repository https://www.kaggle.com/datasets/tawsifurrahman/covid19-radiography-database (accessed on 14 August 2022), which was curated by [93,94]. It is publicly available. Further details can be found here.
